# Robust IMPT and follow-up toxicity in skull base chordoma and chondrosarcoma—a single-institution clinical experience

**DOI:** 10.1007/s00066-024-02280-0

**Published:** 2024-08-29

**Authors:** Vesna Miladinovic, Yvonne L. B. Klaver, Augustinus D. G. Krol, Michiel Kroesen, Berit M. Verbist, Steven J. M. Habraken, Wouter R. van Furth, Ida E. M. Coremans

**Affiliations:** 1 Department of Radiation Oncology, Leiden University Medical Center, Leiden, The Netherlands; 2 Department of Radiology, Leiden University Medical Center, Leiden, The Netherlands; 3 HollandPTC, Delft, The Netherlands; 4 Radiation Oncology, Erasmus MC Cancer Institute, Rotterdam, The Netherlands; 5 Department of Neurosurgery, Leiden University Medical Center, Leiden, The Netherlands

**Keywords:** Proton therapy, Pencil beam scanning, Metallic implants, Bone neoplasms, Intensity modulated proton therapy, Skull base

## Abstract

**Background:**

Chordomas and chondrosarcomas of the skull base are rare, slowly growing malignant bone neoplasms. Despite their radioresistant properties, proton therapy has been successfully used as an adjunct to resection or as a definitive treatment. Herewith, we present our experience with robustly optimized intensity-modulated proton therapy (IMPT) and related toxicities in skull base chordoma and chondrosarcoma patients treated at HollandPTC, Delft, the Netherlands.

**Methods:**

Clinical data, treatment plans, and acute toxicities of patients treated between July 2019 and August 2021 were reviewed. CT and 3.0T MRI scans for treatment planning were performed in supine position in a thermoplastic mold. In total, 21 dose optimization and 28 dose evaluation scenarios were simulated. Acute toxicity was scored weekly before and during the treatment according to the CTCAE v4.0. Median follow-up was 35 months (range 12–36 months).

**Results:**

Overall, 9 chordoma and 3 chondrosarcoma patients with 1–3 resections prior to IMPT were included; 4 patients had titanium implants. Brainstem core and surface and spinal cord core and surface were used for nominal plan robust optimization in 11, 10, 8, and 7 patients, respectively. Middle ear inflammation, dry mouth, radiation dermatitis, taste disorder, and/or alopecia of grades 1–3 were noted at the end of treatment among 6 patients without similar complaints at inclusion; symptoms disappeared 3 months following the treatment.

**Conclusion:**

Robustly optimized IMPT is clinically feasible as a postoperative treatment for skull base chordoma and chondrosarcoma patients. We observed acceptable early toxicities (grade 1–3) that disappeared within the first 3 months after irradiation.

## Introduction

Chordomas and chondrosarcomas of the skull base are rare and slow-growing malignant bone neoplasms that account for less than 0.2 and 0.15% of all intracranial tumors, respectively [1]. According to the World Health Organization (WHO) classification there are several subtypes of these two tumors including conventional, periosteal, dedifferentiated, and poorly dedifferentiated chordoma and grade I, II, and III chondrosarcoma as well as dedifferentiated, mesenchymal, clear cell, and periosteal chondrosarcoma [2]. They are locally aggressive and have low metastasis rates; exceptions are dedifferentiated chordoma and chondrosarcoma as well as grade III chondrosarcoma, which have somewhat higher metastasis rates [3]. Due to the complex anatomy of the skull base region and the proximity of critical structures, complete tumor resection is challenging and cannot be achieved in many patients. Therefore, postoperative radiotherapy is often considered as an adjuvant therapy following surgery to optimize the chances of local control. Although chordomas and chondrosarcomas of the skull base are considered to be relatively radioresistant, they have been successfully treated with high-dose (70–76 Gy) proton therapy with 40–76% and 60–93.6% five-year local control rates, respectively [4–7]. Nowadays, proton irradiation is favored over photon irradiation due to its distinct property of localized high-energy deposition characterized by the Bragg peak, with very rapid dose fall-off thereafter. This allows more efficient and precise high-dose deposition within the target while fully sparing organs distal to the clinical target volume (CTV). Localized energy deposition of protons together with the adjustability of proton beams for irradiating the tumor per layer at different depths renders IMPT a good treatment option for skull base chordomas and chondrosarcomas, which are often adjacent to dose-critical organs and require administration of high doses. This technique has been used for over 20 years now; however, reports on its application in the treatment of skull base tumors, in particular chordomas and chondrosarcomas, are scarce, owing to the rarity of these two malignancies [4, 8–11].

Herewith, we present our single-institution experience with robustly optimized intensity-modulated proton therapy (IMPT) and related toxicities in skull base chordoma and chondrosarcoma patients.

## Materials and methods

### Patient inclusion

All patients with histopathologically confirmed chordoma and chondrosarcoma of the skull base treated with IMPT between July 2019 and August 2021 in HollandPTC (Delft, the Netherlands) were included in the analysis. Clinical data, treatment planning details, and acute toxicity data of these patients were retrospectively analyzed. This study was approved by the local medical ethics committee (study reference number G20.069). Included patients have signed the consent form for use of their data for research purposes.

### Patient simulation and immobilization

Computed tomography (CT) and 3.0 T magnetic resonance imaging (MRI) examinations for treatment planning were performed in supine position in a thermoplastic mold (Qfix, Avondale, PA, USA). CT scans (Siemens Healthineers, Erlangen, Germany) were acquired with a 120-kVp energy setting, 3 mm slice thickness, 1 s rotation time, and 600-mm reconstruction diameter over a 512 × 512 image matrix. The MRI (Philips, Best, the Netherlands) imaging protocol consisted of T1 fast field echo (FFE), T1 turbo spin echo (TSE), T2 TSA mDIXON, diffusion-weighted (DW) TSE, fluid-attenuated inversion recovery (FLAIR), and T1 map turbo field echo (TFE) fractional anisotropy (FA) 5 and 15 sequences, including gadolinium-enhanced dynamic T1 TFE and T1 TSE mDIXON sequences. Gadolinium chelate contrast agent (Dotarem®, Guerbet, Villepinte, France) was administered at 0.2 mL per kilogram of bodyweight with 2 mL/s flow using a Medrad® (Bayer AG, Berlin, Germany) power injector.

### Volume definition, treatment planning, and dose prescription

Depending on the stability of the patient’s neck, if needed, stabilization surgery was performed in consultation with the treating neurosurgeons prior to or following the proton therapy. If the stabilization surgery was performed after the proton therapy, patients wore a hard cervical collar for additional neck stability and support; patients did not wear the collar during the radiation sessions. If stabilization surgery could be performed before the proton therapy, the treating neurosurgeons were consulted both prior to and after the surgery to align the use of surgical implants and the clinical target volume in order to optimize the proton treatment plan. Intersection of the proton bundle and the implant was avoided as much as possible by choosing the optimal beam orientation. Treatment planning and optimization were performed with RayStation software (version 10B, RaySearch Laboratories, Stockholm, Sweden) using the fused CT and MRI scans. Gross tumor volume (GTV), the low- and high-dose clinical target volume (CTV), and organs at risk (OARs) were delineated by the treating radiation oncologist. The low-dose CTV was treated to a total dose of 59.5 Gy (RBE) in chondrosarcomas and 59.2 Gy (RBE) in chordomas, and the GTV and high-dose CTV was treated with 70 Gy (RBE) in chondrosarcomas and 74 Gy (RBE) in chordomas. The dose was delivered with three or four beams in 35 fractions for the total high dose of 70 Gy (RBE) and in 37 fractions for the total high dose of 74 Gy (RBE). Robust plan optimization was performed by indicating the physical composite objective for each region of interest (ROI) within the RayStation built-in treatment optimization functionality. Patient alignment uncertainty was considered isotropic, with 3 mm shifts along each of the three axes (−3 mm, 0 mm, and +3 mm), and stopping power prediction uncertainties were approximated as universal density uncertainties of −3%, 0%, and +3% for all beams. A total of 21 dose optimization scenarios and 28 dose evaluation scenarios, including a 3-mm shift along intermediate directions, were simulated. Evaluation of robust optimization was based on the work of Korevaar E. et al. [12]. Voxel-wise minimum and voxel-wise maximum dose, representing the composite of all the minimal and maximal dose values per voxel, respectively, were used for evaluation of the dose distribution.

Treatment was delivered with the Varian Probeam pencil beam scanning proton system (Varian Medical Systems, Palo Alto, CA, USA) in supine position. Positioning cone-beam CT was performed in the first week of the treatment, followed by weekly position evaluation CT scans and daily position verification orthogonal kV-kV imaging.

### Data analysis

Acute toxicity was scored weekly during the treatment and at the end of treatment according to the Common Terminology Criteria for Adverse Events (CTCAE) v4.0 scale. Median follow-up was 35 months (range 12–36 months).

## Results

**Fig. 1:**
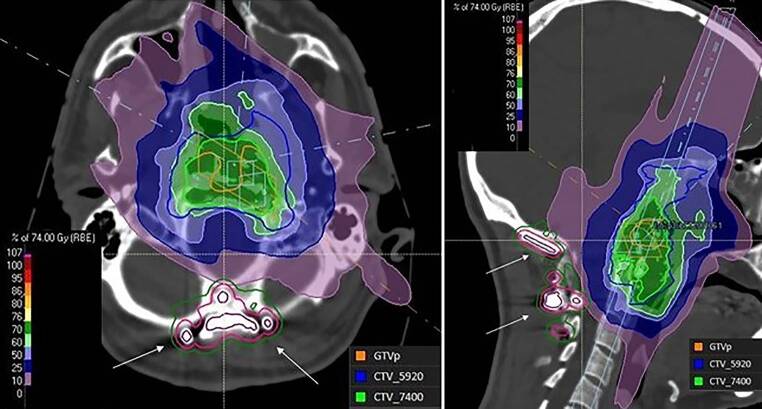
Treatment plan (in transversal and sagittal plains) of a patient with dedifferentiated chordoma located in the petrous apex with titanium implants (*white arrows*). Gross tumor volume (GTV) and clinical tumor volume (CTV) high dose of 70 Gy (RBE) was delivered in 37 fractions using four beams with gantry angles of 70°, 80°, 125°, and 295°

A total of 12 patients (4 males and 8 females) were included in the analysis: 8 patients were diagnosed with a primary chordoma (CH), 1 with a primary dedifferentiated chordoma (DCH), and 3 with a primary chondrosarcoma grade 2 (CSII). One CSII patient was also diagnosed with Ollier’s disease. Median age at diagnosis was 51 years, with a range of 25–70 years. All patients had had 1 to 3 resections prior to the proton therapy, and 4 of them had titanium implants (Fig. 1). Detailed information is given in Table 1. Four patients experienced postoperative complications. One CH patient developed meningitis, lost tongue movement ability, and had difficulties swallowing; a second CH patient also developed swallowing difficulties; a third CH patient had liquor leakage. One CSII patient reported chewing difficulties. Median time between last surgery and first irradiation fraction was 127 days (range 76–677 days).

**Table 1:** Clinical and surgical data of the 12 included patients

Parameter			Total (*n* = 12)	Chordoma (*n* = 8)	Dedifferentiated chordoma (*n* = 1)	Chondrosarcoma grade II (*n* = 3)
Age at diagnosis	*Median, years (range)*		51 (25–70)	51.5 (25–70)	53	42 (37–55)
Gender	*Male (%)*		4 (33.3)	3 (62.5)	1 (100)	0
	*Female (%)*		8 (66.7)	5 (37.5)	0	3 (100)
Bone of origin	*(n)*		Clivus (4)	Clivus (3)	Clivus (1)	Petroclival (3)
			Petroclival (4)	Petroclival (1)		
			Craniocervical junction (4)	Craniocervical junction (4)		
GTV (post-resection)	*Mean, cc (range) *		14.9 (0.82–48.7)	15.4 (0.8–48.7)	39.4	5.3 (1.2–8.8)
Number of surgeries and surgical margins	*1*	*R2*	12	8	1	3
	*2*	*R2*	2	3	0	0
	*3*	*R2*	1	1	0	0
Fixation material	*Titanium*		4	3	1	0
	*None*		8	5	0	3

A cumulative dose of 70 Gy (RBE) for all chondrosarcoma patients and 74 Gy (RBE) for all chordoma patients was administered to the CTV in 2 Gy (RBE) per fraction. Median nominal coverage (at least 98.0% of the volume) of the 59.5 Gy (RBE) low-dose CTV was 99.15% (range 94.39–99.97%) and of the 59.2 Gy (RBE) low-dose CTV was 98.77% (range 91.45–99.96%). Median nominal coverage (at least 98.0% of the volume) of the 70 Gy (RBE) high-dose CTV was 97.66% (range 97.52–99.43%) and of the 74 Gy (RBE) high-dose CTV (at least 98.0% of the volume) was 98.00% (range 83.20–98.99%). Voxel-wise minimum dose coverage for the 59.5 Gy (RBE) and the 59.2 Gy (RBE) low-dose CTV was 94.39% (range 90.49–95.68%) and 94.72% (range 76.28–99.82%), respectively, and for the 70 Gy (RBE) and 74 Gy (RBE) high-dose CTV was 99.98% (range 68.21–100%) and 99.08% (range 63.13–100%), respectively. For 11 patients treatment was delivered with four beams and for one patient with three beams.

Brainstem core and surface and spinal cord and surface were used for nominal plan robust optimization in 11, 10, 8, and 7 patients, respectively. Median nominal dose and voxel-wise maximal dose administrated to the OARs are given in the Table 2.

**Table 2:** Proton plan evaluation and optimization—organs at risk (OARs)

Organ at risk		Median, Gy (RBE), (range)	Used for optimization (Total *n* = 12)	Used for robust optimization (Total *n* = 12)
Brainstem core (D_0.03cc_)	Nominal plan	49.98 (0.00–56.01)	11	11
	Voxel-wise max plan	54.78 (0.00–60.50)	9	9
Brainstem surface (D_0.03cc_)	Nominal plan	54.48 (0.00–59.88)	10	10
	Voxel-wise max plan	61.56 (0.00–66.76)	8	8
Spinal cord surface	Nominal plan	38.40 (0.00–58.66)	8	7
	Voxel-wise max plan	55.48 (0.00–62.67)	9	8
Spinal cord core	Nominal plan	39.05 (0.00–55.12)	9	8
	Voxel-wise max plan	51.34 (0.00–59.81)	9	8
Optic chiasm	Nominal plan	25.92 (0.00–55.47)	11	4
	Voxel-wise max plan	36.11 (0.00–59.73)	11	4
Optic nerve R	Nominal plan	29.94 (0.00–57.13)	10	4
	Voxel-wise max plan	40.83 (0.00–62.97)	9	4
Optic nerve L	Nominal plan	24.54 (0.00–54.39)	10	4
	Voxel-wise max plan	31.45 (0.00–60.70)	9	4
Hippocampus R (V_40_)	Nominal plan	8.90 (0.00–38.23)	10	0
Hippocampus L (V_40_)	Nominal plan	10.66 (0.00–35.09)	8	0
Brain—CTV (D_max_)	Nominal plan	66.62 (0.00–70.34)	8	5
Cochlea R (D_mean_)	Nominal plan	31.82 (0.00–66.44)	9	0
Cochlea L (D_mean_)	Nominal plan	5.82 (0.00–47.19)	8	0
Pituitary (D_mean_)	Nominal plan	10.09 (0.00–55.66)	7	0

*L *left, *R *right, *D0.03cc* highest dose in 0.03cc of OAR volume, *V*_40_ volume receiving 40Gy (RBE) or more, *D*_max_ maximal dose, *D*_mean_ mean dose

Treatment was administrated in 35 fractions to 3 patients and in 37 fractions to 8 patients. One patient experienced a severe headache following the first fraction, due to which the treatment was paused, and a ventriculoperitoneal drain was placed. As the disease progressed during this pause, treatment plan adaptation was performed for this patient; treatment was successfully continued without interruption thereafter.

The most frequently observed pretreatment complaints were grade 1 or 2 hearing impairment (4 patients), extraocular muscle paresis (3 patients), optic nerve disorder (2 patients), and swallowing problems (3 patients). Acute toxicities at the end of treatment were limited: one patient developed grade 4 hearing impairment and one patient developed grade 3 speaking disorder. Most of these toxicities disappeared spontaneously within 3 months following treatment, as presented in Table 3. Only one patient developed grade 2 dry eye 3 months following the treatment, which gradually disappeared 1 year after the treatment. One patient had grade 1 dry mouth for a year following the treatment, and 2 patients had grade 1 extraocular muscle paresis, which persisted along the follow-up course; all other toxicities have disappeared. One year following the clival chordoma treatment, one patient was treated with proton therapy and surgery for another chordoma lesion in the lumbar spine. Two patients with rapidly progressive chordomas developed metastasis and died 1 and 3 years following the treatment. One patient reported blurred vision >2 years after the proton therapy; the complaint was caused by a recurrent chordoma occurring in the low-dose CTV 14 months following proton therapy, which, over time, slowly grew, pressing on the third cranial nerve.

**Table 3:** Frequencies of complaints at inclusion and follow-up. Toxicity grades scored among the 12 reported patients according to the CTCAE v4.0 scale

	Grade	Pre-PT	End of PT	3 months post-PT	6 months post-PT	1‑year post-PT	2 years post-PT	>2 years post-PT
		*n *(%)	*n* (%)	*n* (%)	*n* (%)	*n* (%)	*n* (%)	*n* (%)
Middle ear inflammation	1	1 (8.33), patient 6	2 (16.67), patients 9 and 12	0	0	0	0	0
	2	0	1 (8.33), patient 6	1 (8.33), patient 6	0	0	0	0
Hearing impairment	1	3 (25.00), patients 1, 10, and 12	2 (16.67), patients 10 and 12	3 (25.00), patients 1, 5, and 12	0	0	0	0
	2	1 (8.33), patient 6	0	1 (8.33), patient 10	0	0	0	0
	4	0	1 (8.33), patient 1	0	0	0	0	0
Dry eye	1	0	0	0	1 (8.33), patient 3	0	0	0
	2	0	0	1 (8.33), patient 3	0	0	0	0
Extraocular muscle paresis	1	2 (16.67), patients 8 and 9	3 (25), patients 4, 8, and 9	2 (16.67), patients 4 and 9	2 (16.67), patients 4 and 9	2 (16.67), patients 4 and 9	2 (16.67), patients 4 and 9	2 (16.67), patients 4 and 9
	2	1 (8.33), patient 4	0	0	0	0	0	0
Optic nerve disorder	1	1 (8.33), patient 8	0	0	0	0	0	0
	2	1 (8.33), patient 1	1 (8.33), patient 1	0	0	0	0	0
Blurred vision	2	0	0	0	0	0	0	1 (8.33), patient 12
Dry mouth	1	0	2 (16.67), patient 7, 11	1 (8.33), patient 7	1 (8.33), patient 7	1 (8.33), patient 7	0	0
Swallowing problems (dysphagia)	1	2 (16.67), patients 4 and 11	2 (16.67), patients 4 and 11	1 (8.33), patient 11	0	0	0	0
	2	1 (8.33), patient 8	1 (8.33), patient 8	0	0	0	0	0
Radiation dermatitis	1	0	2 (16.67), patients 10 and 11	0	0	0	0	0
Taste disorder (dysgeusia)	1	1 (8.33), patient 1	1 (8.33), patient 1	0	0	0	0	0
	2	0	1 (8.33), patient 9	0	0	0	0	0
Speaking disorder (dysphasia)	1	0	1 (8.33), patient 9	0	0	0	0	0
	2	0	1 (8.33), patient 8	0	0	0	0	0
	3	0	1 (8.33), patient 11	0	0	0	0	0
Alopecia	1	0	3 (25), patients 7, 10, and 11	1 (8.33), patient 11	0	0	0	0

## Discussion

The results presented in this manuscript show that robustly optimized IMPT can be used for high-dose (70–74 Gy [RBE]) treatment of skull base chordomas and chondrosarcomas with good CTV coverage (97.66–98.00%), even in the presence of implants, while sparing the adjacent dose-sensitive structures with little plan adaptation and necessary position-evaluation cone-beam CT, weekly CT scans, and daily kV-kV imaging.

Although gross total resection is considered the mainstay of treatment for chordomas and chondrosarcomas, for lesions localized in the skull base this is not possible without major morbidity for most patients. Dose-escalated radiotherapy is therefore often used as an adjuvant or alternative treatment to optimize local tumor control with good results, particularly for radiotherapy delivered with protons [4–6, 8, 13–21]. Results of one of the largest studies on long-term outcomes of proton therapy in 151 chordomas and 71 chondrosarcomas of the skull base have shown that long-term local tumor control can be achieved regardless of the level of resection [6]. In a subset of patients, the biological behavior of the tumor is such that radiotherapy can be reserved for treating tumor recurrence.

A potential downside of radiation therapy in general that has to be considered is its sensitivity to system setup and patient positioning uncertainties. In photon radiotherapy these uncertainties are accounted for by adding an extra margin around the clinical target volume to create the planning target volume (PTV) [8, 21]. Contrary to photon beams, proton beams are more sensitive to the presence of heterogeneities along their path, which can cause dose perturbations and formation of so-called hot spots and cold spots within and close to the target. This is of utmost importance, especially when high doses are administered in a location with nearby critical structures, such as the skull base. The PTV approach is not able to account for all these heterogeneities, making it a not so ideal method for proton therapy [8, 22]. However, Monte Carlo simulation-based robust optimization, which re-evaluates and re-calculates treatment plan scenarios for different setups for each proton beam, accounts better for uncertainties in IMPT. Although robust optimization is more time consuming compared to conventional PTV-based optimization, it is less sensitive to uncertainties, making it superior to conventional PTV-based optimization [23–25]. Additionally, for smaller CTVs, as often encountered in the skull base region, worst-case-scenario-based robust optimization proved to be better than conventional optimization [14].

The frequent presence of titanium implants in the vicinity of the irradiated tumor volume renders treatment planning more difficult. The high-density titanium implants cause hardening of the CT X‑rays, thus resulting in image artifacts that often make accurate target delineation difficult. These artifacts, along with the increased medium heterogeneity caused by the implant, further interfere with the accuracy of dose calculation, which could result in tumor underdosage and formation of the previously mentioned hot spots and cold spots. To avoid creation of hot spots, Rutz et al. administrated a reduced dose per fraction (1.8 Gy [RBE]) and reported a positive correlation between the presence of metallic implants and local failure in extracranial chordoma patients treated with proton therapy [26]. DeLaney et al. reported a slightly lower local failure rate (35 vs. 38%), proposing that the location of the tumor and the level of resection have an impact on the local failure rate [27]. Staab et al. reported a 30% 5‑year local control rate in skull base, spinal, and sacral chordoma patients with metallic implants treated with proton therapy [28]. Difficulties with artifacts, dose calculations, and beam positioning that arise are a main reason why some centers opt not to perform proton therapy in patients with implants [29]. Nevertheless, in our study, 4 patients with titanium implants were successfully treated with IMPT. In our center, the main strategy for administrating proton therapy in such cases is consultation with the treating neurosurgeons both prior to and after the surgery in order to align the position of the surgical implant and clinical target volume for the optimal proton treatment plan.

With the low radiosensitivity of chordomas and chondrosarcomas, administration of the necessary high doses in the vicinity of critical organs, especially in the complexly structured area of the skull base, can be challenging. Unlike photons which continuously deposit energy along their trajectory, protons have a rapid dose fall-off at the end of their trajectory, thus allowing locally high dose deposition while sparing the adjacent dose-sensitive organs and rendering proton therapy a more favorable treatment modality. Reported 5‑year local control rates are in favor of skull base chordoma and chondrosarcoma patients treated with protons compared to those treated with photons—81 and 65.3% in chordomas and 94 and 88.1% in chondrosarcomas, respectively [30, 31]. Despite being more sensitive to uncertainties as compared to photon therapy, as mentioned above, proton therapy-induced toxicities are acceptable. Ares C. et al. reported grade 3 toxicities in 3 patients (neuropathy, central nervous system necrosis) and grade 4 toxicity in only 1 patient (neuropathy) among 64 patients during a mean follow-up period of 38 months [30]. Mattke M. et al. reported up to grade 2 temporal lobe toxicity in 44 out of 147 skull base chordoma patients treated with proton therapy, out of whom 20 patients had temporal lobe necrosis [11]. Other commonly reported early toxicities such as hearing impairment, dry eye, vision impairment, dry mouth, and radiation dermatitis were also seen among the patient group presented in this study (Table 3; [7, 32–35]). Most of these toxicities spontaneously disappeared 3 months after treatment.

Our results, as well as those of previously published studies, demonstrate that robustly optimized IMPT is feasible for lesions located in the head and neck region with low-grade toxicities [10, 11, 15, 24, 33]. Long-term sequelae are currently actively monitored and recorded for future treatment adaptation and improvement.

## Limitations

The main limitations of this study comprise the small group of patients analyzed, which is inherent to the rareness of the disease, and the relatively short follow-up period. Because treatments for this disease in HollandPTC started in 2019, long-term follow-up is not yet robust enough to draw conclusions. Therefore, this study focuses on acute toxicity and reporting the treatment technique. Patients are included in a long-term follow-up program to prospectively collect data on effectiveness and long-term toxicity. Another limitation is the unavailability of results on patients treated with conventional photon therapy as a control group. In the Netherlands, treatment of skull base chordomas is considered a standard indication for proton therapy [36], so all patients with this disease are referred for and treated with proton therapy. A recent photon cohort to compare to is not available.

## Conclusion

Robustly optimized IMPT is clinically feasible as an adjuvant treatment in patients diagnosed with skull base chordoma and chondrosarcoma with acceptable early toxicities of grade 1 up to grade 3 that tend to disappear in the first 3 months after irradiation. In the presence of surgical implants, close collaboration between neurosurgeons and radiation oncologists is key to adequate treatment planning.

## Abbreviations

CTV: Clinical target volume
GTV: Gross tumor volume
IMPT: Intensity-modulated proton therapy
OARs: Organs at risk
PT: Proton therapy
RBE: Relative biological effectiveness
